# Supplementary material for: Netcombin: An algorithm for constructing optimal phylogenetic network from rooted triplets
Source: PLoS One. 2020 Sep 18;15(9):e0227842. doi: 10.1371/journal.pone.0227842 (PMC7500971; doi:10.1371/journal.pone.0227842)
Supplement: S4 File — (ZIP) [file pone.0227842.s004.zip › SequenceData-Triplet/sequenceData-10-20-40-Triplet2/data-(sample 10- length 500-num seq 20-m rate 10^-7)/5 seq.pdf]

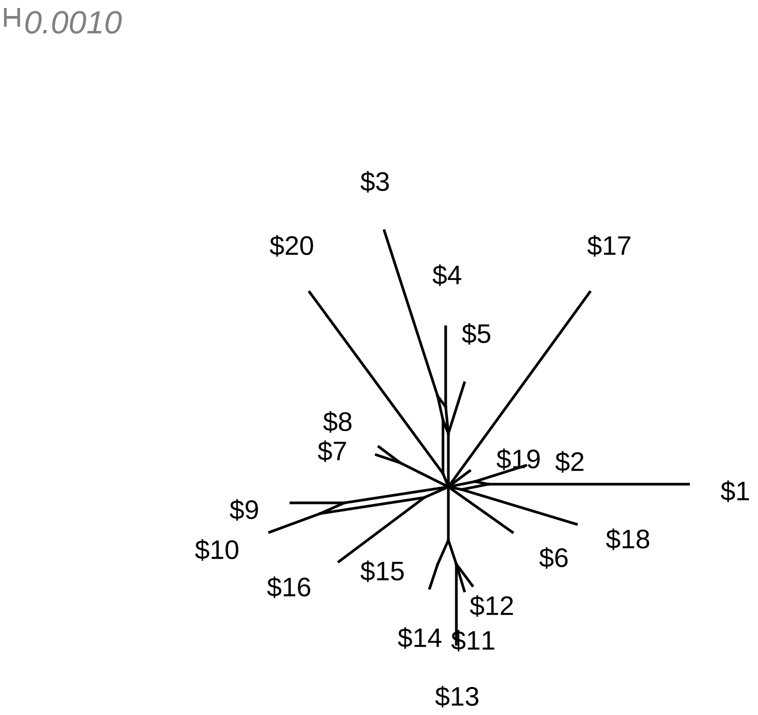
0.0010
$3
$20
$17
$4
$5
$8
$7
$19
$2
$1
$9
$10
$18
$6
$15
$16
$12
$14
$11
$13
